# Determinants of hardship financing in coping with out of pocket payment for care seeking of under five children in selected rural areas of Bangladesh

**DOI:** 10.1371/journal.pone.0196237

**Published:** 2018-05-14

**Authors:** Tazeen Tahsina, Nazia Binte Ali, Md. Abu Bakkar Siddique, Sameen Ahmed, Mubashshira Rahman, Sajia Islam, Md. Mezanur Rahman, Bushra Amena, D. M. Emdadul Hoque, Tanvir M. Huda, Shams El Arifeen

**Affiliations:** 1 Maternal and Child Health Division (MCHD), icddr,b, Dhaka, Bangladesh; 2 Department of Economics, George Washington University, Washington DC, United Sates of America; 3 Nobokoli Program, World Vision, Dhaka, Bangladesh; Brown University, UNITED STATES

## Abstract

**Background:**

Around 63% of total health care expenditure in Bangladesh is mitigated through out of pocket payment (OOP). Heavy reliance on OOP at the time of care seeking poses great threat for financial impoverishment of the households. Households employ different strategies to cope with the associated financial hardship.

**Objective:**

The aim of this paper is to understand the determinants of hardship financing in coping with OOP adopted for health care seeking of under five childhood illnesses in rural setting of Bangladesh.

**Methods:**

A community based cross sectional survey was conducted during August to October, 2014 in 15 low performing sub-districts of northern and north-east regions of Bangladesh. Of the 7039 mothers of under five children surveyed, 1895 children who suffered from illness and sought care for their illness episodes were reported in this study. Descriptive statistics and ordinal regression analysis were conducted.

**Results:**

A total number of 7,039 under five children reported to have suffered illness by their mothers. Among these children 37% suffered from priority illness. Care was sought for 88% children suffering from illnesses. Among them 26% went to a public or private sector medically trained provider. 5% of households incurred illness cost more than 10% of the household’s monthly expenditure. The need for assistance was higher among those compared to others (31% vs 13%). Different financing mechanisms adopted to meet OOP are loan with interest (6%), loan without interest (9%) and financial help from relatives (6%) Need for financial assistance varied from 19% among households in the lowest quintile to 9% in the highest wealth. Ordinal regression analysis revealed that burden of hardship financing increases by 2.17 times when care is sought from a private trained provider compared to care seeking from untrained provider (CI: 1.49, 3.17). Similarly, for families that incur a health care expenditure that is more than 10% of their total monthly expenditure (CI:1.46, 3.88), the probability of falling into more severe financial burden increases by 2.4 times. We also found severity of the hardship financing to be around half for households with monthly income of more than BDT 7500 (OR = 0.56, CI: 0.37, 0.86). The burden increased by 2.10 times for households with a deficit (CI: 1.53, 2.88) between their monthly income and expenditure. The interaction between family income and severity of illness showed to significantly affect the scale of hardship financing. Children suffering from priority illness belonging to poor households were found have two times (CI: 1.09, 3.47) higher risks of suffering from hardship financing.

**Conclusion and policy implications:**

Findings from this study will help the policy makers to identify the target groups and thereby design effective health financing programs.

## Background

Bangladesh has experienced a dramatic decline in under-five mortality in the last four decades [1] despite facing innumerable challenges and adversities. Apart from improved access to and use of health services, focus on women’s education and empowerment contributed to the reduction in child mortality [2]. Out of pocket payments are used as a means to complement government expenditure and financial protection in health care. Many households suffer financial hardship, or are even impoverished because their members pay from pocket at the time they receive health care [3]. Globally, an estimated 150 million people endure severe financial hardship and 100 million are pushed below poverty line each year as they need to use and pay for health care[3]. It is important to investigate whether households in Bangladesh suffer from financial hardship while paying for child health care services. Understanding OOP expenditure and the key factors driving the burden of borrowing & selling of asset can help policy makers recognize the intensity of the problem and adapt likely policy responses [4–5].

High OOP and low government spending characterize healthcare financing in Bangladesh. Per-capita total health spending in Bangladesh in 2012 was US$27 while households contribute almost 63% of total health care expenditure [6]. OOPs have shown an increasing trend in recent years. OOPs for child care occur frequently which are significant [7]. Sometimes these costs are sudden and unexpected [8]. More than 30 percent of people lived below the poverty line (i.e. below $PPP1.25 per person per day) in 2010 [9]. Fertility rate among the poor is higher while children from the poorer households are more prone to suffer from illness. Ensuring financial risk protection when seeking health care is key to achieving Universal Health Coverage. The Health, Population and Nutrition Sector Plan (HPNSP) 2017–2022 aims to achieve Universal Health Coverage (UHC) ensuring equity, quality and efficiency. Protecting households from financial catastrophe and impoverishment is a key to achieve UHC goal [10–11].

Heavy reliance on OOP poses financial burden on households and leads to different mechanisms to be adopted to mitigate necessary payment for healthcare for under-five child’s illness. Sometimes the coping strategies associated to OOP can be “impoverishing” if household is faced with welfare loss and pushed further to a lower wealth/income status. Coping mechanisms adopted based on the extent of financial burden and thus depends on the type of care sought, duration of illness and the level of facility utilized [7]. Private providers charge higher service fees than public providers for patients of all ages in low income countries [12–13]. Sometimes patients pay unofficial (‘under the table’) user fees at the public health facilities in many settings [14]. OOPs may differ in terms of chronic conditions of the patients and length of illness [15].

Many households in low income countries do not have access to any social health protection schemes and therefore rely on existing resources or borrow money or in worst possible scenario sell off their assets [15]. It is known that high OOP leads to high financial burden on families. However, it is critical to understand how this OOP is being mitigated and what are the triggering factors that drag households into hardship financing.

Coping mechanisms provide information on how households respond to OOPs and how these may affect their future welfare status [15]. Households adopt different strategies in the face of health expenditures [16–18]. These strategies include: use savings, sale of assets and livestock, borrow money from friends and family, take loan on interest, reduce expenditure on other basic needs including food and discontinue children study at schools. Households’ members arrange additional work to repay loan and to cover lost income [19–20]. There may be differences in the ability of households to cope with financial costs of illness. The burden of financial hardship may endanger vulnerable household livelihoods and push household members to poverty or reduced welfare [21–23].

Costs of illness that households must cover are sensitive to household income [24–27]. This association is even stronger in households of low-income countries with no social health protection mechanisms in place [28]. Households or parents with under-five children are at risk of going through financial hardship when paying for healthcare which may eventually lead to catastrophe. Although, many studies have looked at the level of households’ expenditures and cost of treating under-five children in developing country settings [29–32], there is almost no available information on factors that trigger decision on different strategies for mitigating the costs. There is very little information available on the how choice of public versus private health facilities can influence level of burden due to coping mechanisms for child health care services in developing countries. Impact of hardship financing for under-five children on household living status has received little attention in Bangladesh. This lack of knowledge is an impediment to policy formulation and implementation aimed at reducing the financial burden of child health illness. The current paper will add to this understanding by presenting empirical data to describe the factors affecting the decision to undertake different coping strategies for financing healthcare in the face of under-5 child illness. It will also shed light on what can possibly risk households to fall into deeper financial hardship as a result of the strategy adapted. It will also aide the government with useful information in designing evidence informed health financing scheme targeted to under five children.

## Materials and methods

### Study design and data

The data for this study were obtained from a large community based cross sectional survey conducted in 15 low performing sub districts of greater Mymensingh and northern regions of Bangladesh. This survey was carried out in 2014 as part of the baseline assessment of an integrated nutrition program of World Vision Bangladesh called “Nobokoli’. Nobokoli interventions cover areas of nutrition, water, sanitation hygiene and livelihood to address the underlying causes of undernutrition. The interventions are focused on the first 1,000 days of life from pregnancy till the child is 2 years of age. icddr,b is using a quasi-experimental study design to evaluate the impact of Nobokoli program. The survey covered villages from both intervention and comparison areas.

The survey collected a wide range of information on child health care practices such as feeding, nutritional status, morbidity, care seeking practices and it’s associated out of pocket expenditure (OOP), financing mechanisms adapted for incurring OOP and household sociodemographic indicators.

### Sampling and sample size

The study followed a multistage clustered random sampling technique. At first, 15 sub districts were selected from the 20 Nobokoli intervention areas using probability proportionate to size (PPS) for optimal resource management. In the second stage, 140 clusters were randomly selected from both intervention & comparison areas by PPS where each village with a population of 1,000–1800 was defined as a cluster. Finally, 52 randomly selected mothers of under-five child were interviewed from each cluster. A total of 8,679 households were surveyed and 99.9% consented for the interview. Details of the baseline survey has been published elsewhere [33].

**Fig 1:**
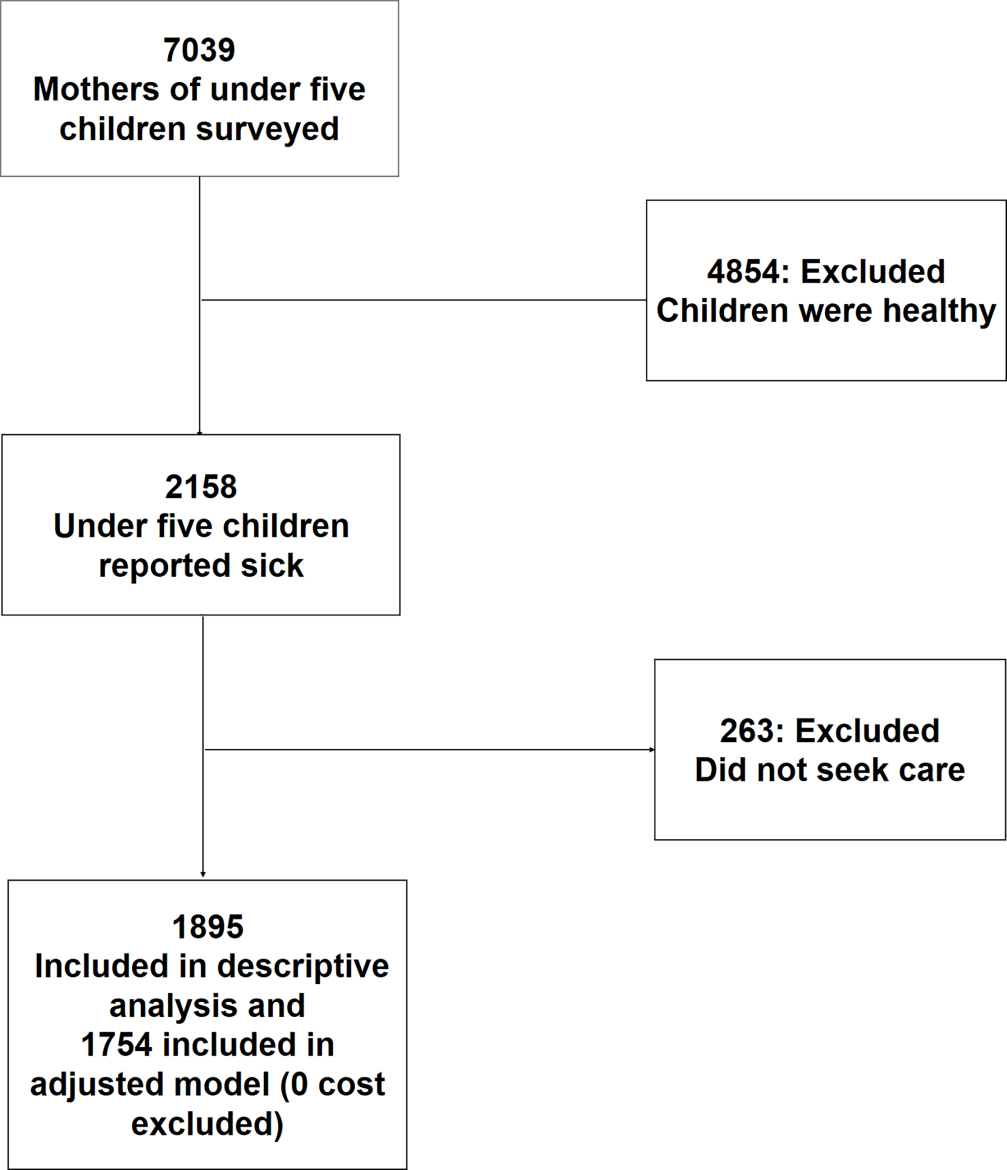
Selection of sample for analysis.

The survey covered 7039 mothers of under five children. Out of which 2158 children suffered from any sort of illnesses in the two weeks prior to survey. Our sample (n = 1895) for this paper includes the children who suffered from illnesses and sought care for their illness episodes (Fig 1)

Inclusion criteria: Children who suffered from illness within two weeks preceding the survey and sought care for their illness. The main objective was to explore the extent of hardship encountered in paying for healthcare expenses.

Exclusion criteria: Those who did not seek care for illness. Fig 1 details out the sample selection criteria.

### Ethical consideration

The Ethics Review Board of icddr,b approved the study and the study followed the principles embodied in the Declaration of Helsinki. Informed written consent were taken from the respondents before starting the interview (PR14044).

### Measurement process & quality assurance

A pretested structured validated questionnaire was administered to mothers of under-five children in national language, Bangla. The questionnaire primarily followed the Bangladesh Demographic and Health Survey 2014. This gave the opportunity to compare final outcome data with national trend. Questions on coping strategies were collected following a pre-used tool in a large community based cluster randomised trial involving more than 35,000 women (Shonjibon Trial #1026864). The tool was developed through expert consultation with health economists and administered on a large sample. Both tools have been validated in Bangla. Reference period for collected information on illness episodes of under-five children, care seeking, OOP and funding mechanisms were two weeks preceding the survey to minimize recall bias. All information collected are reported by mothers. Data collectors experienced in survey data collection was deployed for data collection after 10 days in house training and 2 days field practices. Two tiers of supervisors monitor data collection activity through regular spot checking. A separate quality assurance team conducted 5% of the re-interview to ensure data quality.

#### Variables

The dependent variable was an ordered variable “hardship financing” representing the burden of financing out-of-pocket payments [34–36]. Mothers were asked regarding the means the household had to undertake in the face of any out-of-pocket payments for care-seeking of under-five children over the preceding two weeks due to morbidity. They selected among regular income, savings, help from relatives/friends, loan without interest, loan with interest, sold assets/livestock’s/ personal belongings or mortgage of land or properties. These options were grouped together to create an ordinal scale based on the intensity of burden of financial hardship. Regular income and savings were grouped together considering these incur no burden for hardship financing while selling of assets/livestock’s/ personal belongings or mortgage of land or properties were grouped worst case scenario posing highest threat to household’s financial stability[35]. The scale of dependent variable ranges from 0–4 where 0 is “savings/regular income”, 1 is “took help from relative”, 2 is “took loan without interest”, 3 is “took loan with interest’ and 4 is “sold assets or mortgage lands”.

Explanatory variables include illness type, provider type, socio-economic characteristics of the household and mother’s background characteristics. Illnesses were categorized based on reported symptoms and severity of illness; priority illness (children who had pneumonia, diarrhoea with dehydration, dysentery, malaria, or measles) and non-priority illness (children who had a cough or cold, acute or chronic ear infection, diarrhoea with no dehydration, anaemia, or fever without malaria). This has been adapted from the Multi Country Evaluation (MCE) of Integrated Management of Childhood Illness (IMCI) [37]. Health care providers were classified into untrained health care providers (pharmacy, ayurveda, homeopath and traditional healer etc), public trained (trained provider working in the government health care facilities) and private trained (trained provider working in private health care facilities). Definition of trained and untrained was taken from Bangladesh Demographic and Health Survey 2014 (BDHS) [1]. Mother’s background characteristics include mother’s level of education and employment status. Information on OOP was collected in local currency (Bangladesh Taka) for up to three consecutive visits. OOP variable was constructed by aggregating the direct and indirect costs of care seeking for the patient and opportunity costs for their accompanying caregivers. Illness cost as percentage of total household expenditure was calculated as a proportion of OOP to total monthly expenditure of the household.

Household socio-economic characteristics include monthly income of the household, balance between households last month’s income and expenditure and households wealth status. Household income was collected for preceding one year while expenditure information was collected for the preceding one month. Mother’s overall opinion regarding balance between household income and expenditure was recorded as equal income and expenditure, savings and deficit. Household wealth status was determined by ownership of household durable goods, dwelling characteristics, source of drinking water, sanitation facility, possession of land and domestic animals using principle component analysis. Wealth status was stratified into five quintiles where ‘one’ represented the poorest and ‘five’ represented the richest. Mother’s education was classified as no education, primary incomplete (1–5 years of education) and primary and above (6+ years of education). Mother’s occupation was classified into unemployment, unskilled and skilled employment.

### Statistical analysis

We used Stata 13.0 Special Edition software (StataCorp. 2013. Stata Statistical Software: Release 13. College Station, TX: StataCorp LP) for data analysis. Percent distribution of different sources of financial mechanisms were examined by explanatory variables The next stage of analysis explored the proportion of households who failed to meet OOP for health care and had to seek financial assistance from external sources. Going further down, we looked at details on how the costs incurred for care-seeking were paid by the families and the severity of financial hardship in terms of foregone future income of households. Finally, we developed an ordered scale for measurement of financial hardship for OOP in terms of possible loss in future income. Sale of asset that can bring people down to a lower wealth quintile have been considered to be the most severe consequence followed by loan with interest, loan without interest and loan from relatives. For the categorical explanatory variables, dummy variables were created for regression analysis. Multivariable ordered logistic regression was used to explore the determinants of hardship financing for OOP and the analysis unit was households. Only households that reported care seeking for illnesses of their under five children in the last week of survey data collection were included in the regression model. Explanatory variables found significant in the bivariate analysis were put into the multivariable model. Interaction of the explanatory variables were examined. The basic assumptions of modelling were tested and met. Covariates of risk for hardship financing were reported using odd ratios.

## Results

From the survey, we gathered reports for a total number of 7,039 under five children from their mothers. Around 51% of these mothers had completed at least primary level and more than 12% completed secondary education while 90% were unemployed. The households were equally distributed across wealth quintiles. Median monthly income was Bangladesh Taka (BDT) 7525 Inter Quartile Range (IQR) (5666–11250)_with 32% households having deficit after bearing all expenses.

Among these children, 31% suffered from some illness over the two weeks preceding the survey and 37% of them were suffering from priority illness while the rest were suffering from non-priority illness. Care was sought for 88% children suffering from illnesses. Among them 26% went to a public or private sector medically trained providers (Table 1). Median out of pocket expenditure for care seeking of under five children was 150 (IQR_70–270) BDT. Only 5% households incurred illness cost more than 10% of household’s monthly expenditure.

**Table 1:** Background characteristics of the households having under five children in the rural areas of Bangladesh 2014.

Background characteristics		n = 7039	Percentage (%)
Mother’s Education	No education (0 years)	1,343	19.1
	Primary incomplete (1–4 years)	1,257	17.9
	Primary complete to secondary incomplete(5–9 years)	3,571	50.7
	Secondary complete or higher (10+ years)	868	12.3
Mother’s Employment Status	Unemployed	6,359	90.3
	Unskilled worker	238	3.4
	Skilled worker	442	6.3
Monthly total household income (in taka)	Median (IQR)	7,003	7525 (5666–11250)
Savings status of household after all expenses	Both income and expenditure equal	3,522	50.0
	Savings	1,238	17.6
	Deficit	2,279	32.4
Wealth Quintile	Lowest	1,446	20.5
	Second	1,392	19.8
	Middle	1,438	20.4
	Fourth	1,393	19.8
	Highest	1,370	19.5
Last two week morbidity among under-five children	Any illness	2158	30.7
Type of illnesses (n = 2158)	Nonpriority illness	1,353	62.7
	Priority illness	805	37.3
Care-seeking for illnesses among under-five children (n = 2158)	Care-seeking from any provider	1895	87.8
Type of provider* (n = 1895)	Untrained provider	1,394	73.6
	Trained public provider	235	12.4
	Trained private provider	265	13.98
Out of Pocket Expenditure	Median (IQR)	1895	150(70–270) BDT
Illness cost as percentage share of total household’s monthly expenditure(n = 1895)**	0–10	1788	94.6
	>10	101	5.4

*1 provider missing

**6 cost information missing

**Fig 2:**
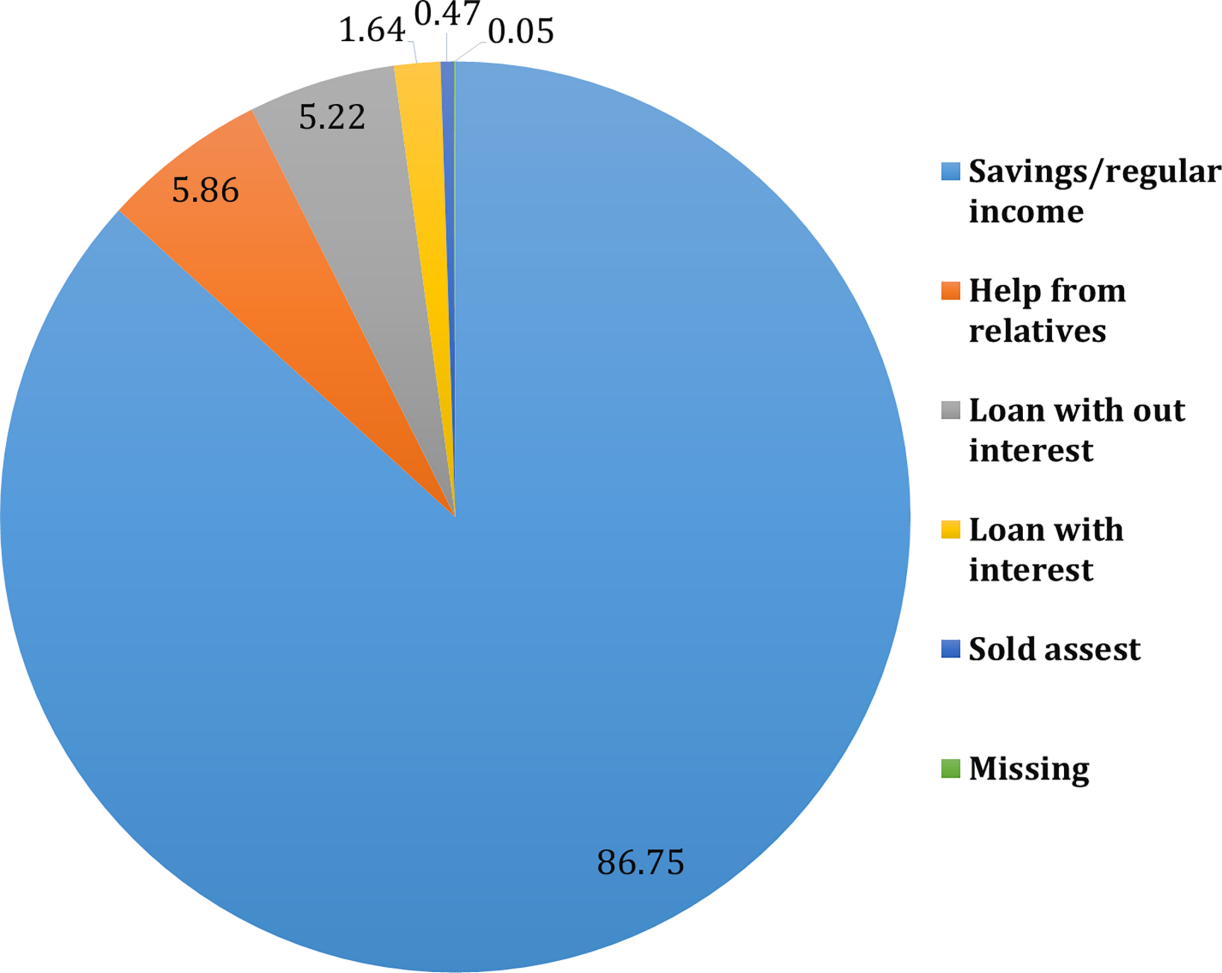
Distribution of different financing mechanisms for care seeking for under five children.

In our study area, more than 13% of the households needed external financial assistance for meeting health care expenditure for care-seeking of children under five years of age. Eighty seven percent of the families who sought care for illness were able to pay from income or savings while 6% took loan from relatives. Five percent had to seek loan without interest while 2% took loan with interest and half a percentage of households had to sell their assets (Fig 2).

Children of families who suffered from a priority illness needed more financial support (16%) from external sources compared to those having non-priority illness (12%). Mothers with secondary or higher level of education took less amount of loan compared to those without any education (1% vs 9%). Among children who were ill and for whom care was sought from trained private health care providers, nearly 6% families took loan with interest, more than 9% took loan without interest and 6% received financial help from their relatives. These rates were higher compared to those who sought care from trained public providers or untrained providers (Table 2).

**Table 2:** The percentage of different financing mechanisms adopted to meet OOP of care seeking for illnesses of under five children by type of illnesses, type of provider and background characteristics, in Bangladesh 2014.

		Number of under-five children who sought care	Different mechanisms for financing OOP				
			% Savings/ households monthly income	% took help from relatives	% took loan without interest	% took loan with interest	% mortgaged or sold assets/land
Overall		1895	86.8	5.9	5.2	1.6	0.5
Type of illnesses	Non-priority illness	1157	88.4	5.2	4.5	1.3	0.5
	Priority illness	738	84.2	6.9	6.4	2.2	0.4
Type of provider	Untrained provider	1394	88.5	5.7	4.5	0.8	0.5
	Public trained provider	235	85.1	6.8	5.5	1.7	0.9
	Private trained provider	265	78.9	6.0	8.7	6.0	0.0
Illness cost as percentage share of total household expenditure *	0–10	1788	87.7	5.7	5.0	1.1	0.5
	>10	101	69.3	9.9	8.9	11.9	0.0
Mother’s Education	No education (0 years)	339	82.9	5.3	9.1	1.8	0.9
	Primary incomplete (1–4 years)	348	83.9	7.8	5.5	2.0	0.9
	Primary complete to secondary incomplete (5–9 years)	976	87.4	5.6	4.7	1.8	0.3
	Secondary complete or higher (10+ years)	232	94.0	4.7	1.3	0.0	0.0
Mother’s Employment Status	Unemployed	1708	86.7	5.8	5.4	1.7	0.5
	Unskilled worker	58	77.6	8.6	10.3	0.0	1.7
	Skilled worker	129	92.3	5.4	0.8	1.6	0.0
Monthly total household income (in taka)**	≤7500	969	83.8	5.9	7.2	2.5	0.6
	>7500	918	89.9	5.8	3.2	0.8	0.3
Savings status after all expenses	Both income and expenditure equal	853	90.5	4.8	4.0	0.5	0.2
	Savings	308	91.2	5.5	1.6	1.0	0.3
	Deficit	734	80.5	7.2	8.2	3.3	0.8
Wealth Quintile	Lowest	396	81.3	6.1	10.1	2.0	0.5
	Second	378	86.0	6.1	4.0	2.7	1.3
	Middle	405	87.7	5.4	5.7	1.2	0.0
	Fourth	385	88.1	6.0	4.4	1.0	0.5
	Highest	331	91.5	5.7	1.2	1.2	0.0

*6 missing

**8 missing

For families that incurred OOP more than 10% of total monthly household expenditure, need for financial assistance was higher compared to that the others (31% vs 13% respectively). The proportion of families who took loan with interest was as high as 12% for those who spent more than 10% of their average monthly expenditure for health care compared to those who spent less than or equal to 10% (1%). Financial burden of health care seeking was lower among mothers who completed at least primary education compared to women with less than primary or no education (11% vs 16% vs 17%). Mothers of sick children who were skilled workers faced lower risk of financial hardship compared to those who were unskilled workers or unemployed (8% vs 22% vs 13%). Households with income of less than BDT 7500 needed more external financial support compared to households with income above this threshold (16% vs 10%), major share of this difference being driven by loan without interest (7% vs 3%). Similarly, households with a monthly deficit after meeting all expenditures face the need for financial assistance (19%) for health care payments than families that end up having some savings (9%). Need for financial assistance varied from 19% among households in the lowest quintile to 9% in the highest wealth quintile. The difference has mostly been driven by the proportion of households taking loan without interest from an external source (Table 2).

Our multivariable ordered logistic regression shows that burden of hardship financing increases by 2.09 times when care is sought from a private trained provider compared to care seeking from untrained provider. Similarly, for families that incur a health care expenditure that is more than 10% of their total monthly expenditure, the probability of falling into more severe debt burden increases by 2.4 times. We also found severity of hardship financing to be around 45% lower for households with monthly income of more than BDT 7500. Mothers who had at least secondary education were less prone (one third) to be faced with hardship compared to mothers with no education. The burden increased by 2.07 times for households with a deficit between their monthly income and expenditure. The interaction between family income and severity of illness showed to significantly affect the scale of hardship financing. Children suffering from priority illness belonging to poor households was found have two times higher risks of suffering from hardship financing (Table 3).

**Table 3:** Determinants of need for hardship financing to meet OOP for care seeking of illnesses among under-five children in Bangladesh 2014.

		Hardship financing to meet OOP			
		Unadjusted odd ratios (95% CI)	P value	Adjusted odd ratios (95% CI)	P value
Type of illnesses	Non priority illness	Ref		Ref	
	Priority illness	1.43 (1.10,1.87)	<0.05	0.95 (0.66, 1.37)	0.78
Type of provider	Untrained provider	Ref		Ref	
	Trained public provider	1.36 (0.92,2.01)	0.13	1.99 (1.29, 3.06)	<0.05
	Trained private provider	2.18 (1.56,3.06)	<0.000	2.17 (1.49, 3.17)	<0.000
Illness cost as percentage share of total household expenditure (N = 1763)	0–10	Ref		Ref	
	>10	3.23 (2.07,5.03)	<0.000	2.38(1.46, 3.88)	<0.000
Mother’s Education	No education (0 years)	Ref		Ref	
	Primary incomplete (1–4 years)	0.91 (0.61,1.35)	0.63	1.07 (0.70, 1.64)	0.75
	Primary complete to secondary incomplete (5-9years)	0.68 (0.49,0.96)	<0.05	0.84 (0.58, 1.22)	0.36
	Secondary complete or higher (10+ years)	0.30 (0.16, 0.55)	<0.000	0.39 (0.20, 0.78)	<0.05
Mother’s Employment Status	Unemployed	Ref		Ref	
	Unskilled worker	1.89 (1.01, 3.55)	<0.05	1.61 (0.82, 3.14)	0.17
	Skilled worker	0.54 (0.28, 1.04)	0.06	0.61 (0.31, 1.21)	0.16
Monthly total household income (in taka)	≤7500	Ref		Ref	
	>7500	0.57 (0.43, 0.75)	<0.000	0.56 (0.37, 0.86)	<0.05
Savings status after all expenses	Both income and expenditure equal	Ref		Ref	
	Savings	0.91 (0.58, 1.44)	0.70	1.08 (0.66, 1.77)	0.75
	Deficit	2.37 (1.77, 3.17)	<0.000	2.10 (1.53, 2.88)	<0.000
Wealth Quintile	Lowest	Ref		Ref	
	Second	0.71 (0.48, 1.04)	<0.05	0.82 (0.54, 1.22)	0.33
	Middle	0.60 (0.41, 0.89)	<0.05	0.76 (0.50, 1.15)	0.20
	Fourth	0.58 (0.39, 0.86)	<0.05	0.90 (0.58, 1.39)	0.62
	Highest	0.39 (0.25, 0.62)	<0.000	0.73 (0.42, 1.27)	0.26
Interaction of illness type & monthly household income category		2.19 (1.25,3.81)	<0.05	1.94 (1.09, 3.47)	<0.05

## Discussion

Our analysis found that financial help from relatives, loan with or without interest and sale of assets were the commonly adopted coping mechanisms to mitigate medical expenses. Severity of financial consequences resulting into hardship increased when care was sought from private health care providers orchildren had priority illness episodes or belonged to an economically less solvent household and when illness cost exceeded 10% of the household’s monthly expenditure. Socio-demographic factor like economic status, level of education and employment status also influenced choice of coping with health care payments. Families seeking care from private trained providers were more likely to face difficult consequences of bearing these payments.

The study is unique in its focus on the coping strategies and predictors of hardship financing mechanisms for childhood illnesses in two weeks prior to the survey at three levels (illness episode, individual and household level) in Bangladesh. Other studies that explored disease specific coping mechanisms for bearing treatment cost with specific focus on childhood pneumonia, tuberculosis, obstetric care or household level OOP, mostly focused at individual or household level determinants [34–36,38–40]. Little attention has been paid to the predictors of hardship financing mechanisms for childhood illnesses.

One limitation in our analysis is that we captured direct OOP for childhood illnesses which may under represent the actual burden as indirect cost of care seeking was not included in the analysis. Since we primarily aimed to understand the financing mechanisms of direct payments, the results would be less likely to be influenced by indirect costs. Missed opportunity of care seeking might be due to financial constraints. People due to poverty overlook or are incapable of recognizing the need for care due to economic barrier or access [41]. Yet our analysis sheds some light on possible characteristics of those who sought care and faced financial hardship to cope with high out of pocket payment for health care. Such valuable piece of information can lead to possible recommendations for improving care seeking through removal of barriers to finance treatment of childhood illnesses. Our data is also limited to detect any change in lifestyle which may have been a case for households that managed to pay from savings or regular income. Dropping out of school or food insecurity could be results of such borrowing that has not been captured in this analysis.

Financial help from relatives, loan with or without interest and selling assets were among the primary mechanisms adopted to mitigate the medical expenses. In low income countries the common trend indicates that more households in the poorest quintile sell assets or borrow money as opposed to the highest wealth quintile [15]. A study based in Indonesia explored both the direct and indirect costs of health shocks and found that through consumption smoothing, households can self-insure around two thirds of the effects of small health shocks [42]. Borrowing is a common technique of smoothing over expenditure consequences of health shocks. Borrowing money from relatives is safer and poses less risk as it is interest free while borrowing from traditional money lenders with high rates of interest can have detrimental effect on future consumption of households [36–40, 43]. When such facilities have been exhausted or not available, people tend to sell assets as a last resort. Availability of microcredit schemes may encourage the poor to borrow when needed. NGOs providing microcredit have gained a high level of trust in Bangladesh. Some of these organizations are providing micro-insurance programs for health linked to microloans and reliant on the group dynamics of microcredit [44]. However, while these programs are able to smoothen consumption in the short run, it does affect future consumption and welfare loss in the long run. In case of acute disease or failure in repayment, families can face more profound catastrophe with long term consequences.

Better preventive mechanisms focusing on child health and nutrition needs to be undertaken at scale. Studies identified stunting to be more prevalent among children in poorer households. Undernutrition poses a higher risk of disease among children leading to higher health care expenditure and therefore higher chance of households facing financial catastrophe. These children are likely to be trapped into a vicious cycle of poor health and poverty leading to lower productivity over the life course [45]. Introduction of social protection schemes such as cash transfer programs are proven to be successful in reducing under nutrition and improving welfare [46].

As a protection and curative platform, pre-payment systems for health financing such as a mandatory health insurance system could be introduced in order to protect vulnerable groups from the impoverishing effects of health care expenditure. Particular focus needs to be given on the poor, especially children when designing such scheme. A decentralised system of the government through devolved funds would reduce possible consequences to be faced by the children over their life-course. However, the constraints on the expansion of health insurance to uncovered groups, such as the informally employed or the poorest, must be considered. The potential role of private sector providers and insurers in expanding access to care can also be explored.

There needs to be availability of high quality services for management of childhood illnesses through the public sector to make the financing schemes successful. Studies suggest that inaccessibility to public health care facilities, poor availability of medicines and equipment, and perceptions of lower quality diagnosis and treatment drive people away from low cost public facilities. This either increases the use of expensive private sector care where families end up incurring higher out of pocket payment or increase use of untrained informal care providers [47–52]. Our findings also interestingly suggests that households are faced with worse financial conditions when choosing a private health care provider. This clearly indicates, with availability of quality and cheaper or free health care services, households are likely to move to the public sector. Improved overall perceptions of public health care service delivery would lead to less risk of potentially catastrophic health care spending and less likely use of negative coping strategies.

In Bangladesh, 63% of Total Health Expenditure (THE) is paid directly from pocket by individuals [6]. This situation is much worse in rural area where THE from public sources is even lower. Over reliance on OOP for health care is regressive and inequitable in nature. Our disaggregated analysis and other reports indicates persistent inequality in health care payments and absence of an equitable financing mechanism. The burden of managing resources to finance health care increases with declining household income. The Maternal Health Vouchers Scheme has been able to improve facility delivery significantly while reducing OOP [53–57]. However, serious concerns were raised regarding the program’s allocative efficiency and service quality. One social protection scheme “Shasthyo Suroksha Karmasuchi (SSK)”that is currently being piloted has its focus limited to people living below poverty line. The scheme is yet to be evaluated, refined and scaled up bringing those above the poverty under its umbrella and making the program sustainable. In line with this effort, scope of health insurance targeted for priority childhood illnesses are needed to be tested to prevent households from falling into financial catastrophe. Again, improvement in service quality is also essential to make these protection schemes successful. Both service quality and financial protection are two key components for achieving UHC which has been repeatedly emphasised in the National Health, Population and Nutrition Sector Plan [58]. The Health Care Financing Strategy 2012–2032 also emphasizes on ensuring financial protection for those in need and make efficient use of resources [59]. One or multiple well designed and well targeted financial protection schemes together needs to be tested and scaled up for protection against catastrophe specially among children. Our findings can feed information for better program design and implementation and ensure better resource utilization.

## Conclusion

Over-reliance on direct payments at the time people need care is one key barrier to achieve universal health coverage. Evidence on OOP for care seeking of illnesses among under five children and financing mechanism to mitigate these OOP are essential for designing effective interventions to ensure universal health coverage. There are ample evidence that introduction of health insurance decreased OOP in India, Thailand, Viet men, Rwanda etc[60–61]. Aligned with global effort, the health financing strategy of Bangladesh emphasizes on ensuring an equitable payment mechanism for health care [59]. Findings from this study will help the policy makers to identify the target groups and thereby design effective health financing programs.

## Supporting information

S1 Data Dataset includes 135 variables. Total observation of this dataset is 7,039. The variable name started with “q” provides the morbidity information of under-five children; “r” presents the child illness cost. The variable name started with “c” and “d” refers to the information of household characteristics and respondent background respectively.
(DTA)
